# Teachable moments and missed opportunities for smoking cessation counseling in a hospital emergency department: a mixed-methods study of patient-provider communication

**DOI:** 10.1186/s12913-014-0651-9

**Published:** 2014-12-20

**Authors:** Mara Buchbinder, Rachel Wilbur, Diana Zuskov, Samuel McLean, Betsy Sleath

**Affiliations:** Department of Social Medicine, University of North Carolina at Chapel Hill, 333 S. Columbia St., 341A MacNider Hall CB 7240, Chapel Hill, NC 27599 USA; Department of Health Behavior and Health Education, University of North Carolina at Chapel Hill, Chapel Hill, NC USA; Departments of Anesthesiology and Emergency Medicine, University of North Carolina at Chapel Hill, Chapel Hill, NC USA; Division of Pharmaceutical Outcomes and Policy, University of North Carolina at Chapel Hill, Chapel Hill, NC USA

**Keywords:** Smoking cessation counseling, Emergency medicine, Teachable moments, Patient-provider communication

## Abstract

**Background:**

While primary care medical clinics have been the most common setting for the delivery of advice about smoking cessation, the hospital emergency department (ED) is a valuable context for counseling medically underserved tobacco users. We conducted a secondary analysis based on a larger audio-recorded study of patient-provider communication about pain and analgesics in the ED. Within a sample of ED patients with back pain, the purpose of this mixed-methods study was to examine how physicians and nurse practitioners capitalize on “teachable moments” for health education to offer spontaneous smoking cessation counseling in the ED.

**Methods:**

Patients presenting to an academic ED with a primary complaint of back pain were invited to participate in a study of patient-provider communication. Audio-recorded encounters were transcribed verbatim. Two coders reviewed each transcript to determine whether smoking was discussed and to build a corpus of smoking-related discussions. We then developed inductively generated coding categories to characterize how providers responded when patients endorsed smoking behavior. Categories were refined iteratively to accommodate discrepancies.

**Results:**

Of 52 patient-provider encounters during which smoking was discussed, two-thirds of the patients indicated that they were smokers. Providers missed opportunities for smoking cessation counseling 70% of the time. Eleven encounters contained teachable moments for smoking cessation. We identified four primary strategies for creating teachable moments: 1) positive reinforcement, 2) encouragement, 3) assessing readiness, and 4) offering concrete motivating reasons.

**Conclusions:**

Most providers missed opportunities to offer teachable moments for smoking cessation. In encounters that contained teachable moments, providers employed multiple strategies, combining general advice with motivation tailored to the patient’s particular circumstances. Creating motivational links to enhance smoking cessation efforts may be possible with a minimal investment of ED resources.

## Background

Cigarette smoking is the leading preventable cause of disability and death in the United States [1]. One in five deaths are attributable to smoking [2,3] and smoking has been linked to a range of chronic health conditions, from cardiovascular disease to cancer [4]. Counseling from a health care provider is an important mechanism for reducing smoking rates [5], and even brief advice to quit increases cessation rates [6,7]. Experts have recommended that physicians follow the “5As” approach to smoking cessation counseling: 1) **A**sk about smoking behavior 2) **A**dvise to quit using clear, strong, and personalized language 3) **A**ssess willingness to stop and motivations to quit 4) **A**ssist by providing strategies and resources 5) **A**rrange follow up [8].

While primary care medical clinics have been the most common setting for the delivery of advice for smoking cessation [7], the emergency department (ED) has also been identified as a key arena for tobacco control efforts [9]. Approximately 130 million ED visits occur annually in the US [10]. Furthermore, ED patients typically smoke at higher rates than the general population and often have limited access to primary care [9-13]. According to a 2011 CDC report, nearly 80% of adult patients who visit the ED lack an alternative care provider [14]. The ED is thus a valuable context for offering advice about smoking cessation, particularly to tobacco users who are medically underserved.

There are multiple barriers to implementing smoking cessation counseling in the ED, including provider level factors (e.g. lack of time, inadequate educational resources) and patient level factors (e.g. a lack of motivation for behavior change) [15,16]. A study of ED physicians’ attitudes toward smoking cessation counseling found that many ED providers believe that the ED is not the appropriate venue for advice to quit smoking and that ED counseling is ineffective [17]. This may explain why only 27% of physicians surveyed in this study reported that they routinely advised smokers to quit. Although evidence of the long-term efficacy of smoking cessation counseling in the ED has been limited [18-22], one recent study found that, in the short-term, ED-based counseling for smoking cessation was as effective as counseling performed in an outpatient clinic [23].

In both ED and primary care settings, much of the literature on patient-provider communication about smoking is situated within targeted smoking cessation interventions [19,23-26]. Fewer studies have described how ED providers offer smoking counseling spontaneously, within ongoing health care interactions [27]. Audio-recording medical visits represents an important method for understanding how communication about smoking unfolds in a naturalistic setting [28], and prior audio-recorded studies of patient-provider communication in the ED have yielded important insights regarding the nature and quality of ED communication, including its constraints [29-31]. The original purpose of the study was to examine communication about pain and analgesics using audio-recordings of patient-provider communication in a hospital ED. For this article, we conducted a secondary analysis of communication about smoking behavior during the history-taking portion of the ED encounter. Specifically, we examined how physicians and nurse practitioners capitalize on “teachable moments” for health education to offer spontaneous smoking counseling to back pain patients in the course of routine care.

### Theoretical framework: teachable moments for health education

The concept of “teachable moments” provides a theoretical framework for our investigation of smoking counseling during ED visits. Although the concept has been employed somewhat inconsistently in the public health literature, most studies have used it to represent an opportunity to facilitate education and behavior change [32]. The concept builds on theories of health behavior—such as Hochbaum’s health belief model [33] and social cognition theory [34]—which emphasize that motivational cues provide an important impetus for health behavior change. Cohen et al. suggest that teachable moments encompass three primary elements: 1) a salient health behavior concern, 2) the deployment of a motivational link between this concern and the possibility of change, and 3) a commitment expressed by the patient toward changing this behavior [35].

Teachable moments for smoking counseling have been linked to specific ecological contexts such as hospitalization [25,36] or pregnancy [37], when patients may be more primed to accept that smoking has negative repercussions for health. Clinic visits for reproductive healthcare, asthma management, dental care, and well child care have all been proposed as effective sites for teachable moments for smoking cessation [38]. Although the ED has been proposed as an important context for teachable moments for injury prevention [39], only two studies, to our knowledge, have examined the ED as a potential site for teachable moments about smoking cessation; [23,40] neither used audio-recorded data to examine patient-provider communication about smoking and to describe how teachable moments might be facilitated.

Much of the literature on teachable moments focuses on opportunities for healthcare providers to actively utilize teachable moments through targeted interventions [41-43]. In contrast, our approach builds on Cohen and colleagues’ investigation of naturally occurring teachable moments [35]. We also draw on Lawson and Flocke’s interactional model of teachable moments [32], which acknowledges that teachable moments may be co-constructed within the unfolding medical encounter. However, our understanding of teachable moments is somewhat broader in scope than that employed by Cohen et al. [35], insofar as we do not think that patients must have expressed an explicit concern about smoking in order for health care providers to deploy a motivational cue toward health behavior change (i.e. smoking cessation), nor do we think that a patient must commit to behavior change in order for the teachable moment to be worthwhile. Therefore, in this paper, we define a teachable moment as a portion of the medical encounter in which a clinician responds to patient self-report of smoking (or other negative health behaviors) by offering motivation, encouragement, and verbal support for health behavior change.

## Methods

The present study is a secondary analysis nested within a larger cross-sectional, observational study of patient-provider communication about back pain and analgesics in an academic ED conducted over an 8 month period during 2012–2013. Back pain was selected as a target of study because it is prevalent in the ED [44] and a leading cause of functional disability [45]. Therefore, within a sample of ED patients with back pain, the purpose of this mixed-methods study was to characterize patient-provider communication about smoking. The study received approval from the Institutional Review Board of the University of North Carolina at Chapel Hill.

### Participants and recruitment strategy

Potential participants were identified using the electronic medical record and screened prior to enrollment for the following eligibility criteria: 1) age 18 or older; 2) English speaking; 3) ability to read the consent form; 4) indicate back pain as a primary complaint. Patients were excluded from the study if they were: 1) unconscious or disoriented; 2) immobilized using a backboard; 3) febrile; or 4) receiving dialysis. Patients who met initial screening criteria were approached in the examination room by the Principal Investigator (PI) or a research assistant (RA), who explained study procedures and obtained informed consent. Patients were invited to participate in an audio-recorded study of patient-provider communication in the ED but were not informed that communication about pain and analgesics was a specific focus of the study. Patients received a $25 gift card for participating in the study.

Patients were clustered within providers, which included attending physicians, medical residents, and nurse practitioners. Providers were recruited using a combination of methods. At the beginning of the study, the PI attended a meeting of the Emergency Medicine department to present the study, which was described as an investigation of patient-provider communication in the ED. Providers were invited to sign a consent form to keep on file in case a future patient was determined to be eligible and willing to participate. Due to low attendance at staff meetings, providers were also emailed a one-page document explaining the study’s goals prior to each data collection shift. The PI or RA then followed up with providers in person to review the study protocol and obtain informed consent. A patient-provider dyad could only participate if both parties agreed. Thus, back pain patients who fit the study’s eligibility criteria but were not assigned to a willing provider were not approached to participate. Providers received a $5 Starbucks gift card each time an encounter was audio-recorded. At the end of the study, providers received a debriefing form that explained that the study sought to characterize communication about pain and pain medications in the ED.

### Data collection

The PI and five RAs completed the data collection procedures over an eight-month period between September 2012 and April 2013. Five-hour research shifts were assigned by the PI, in consultation with the schedules of participating providers, with the goal of balancing daytime, nighttime, weekday, and weekend shifts. After enrolling in the study, patients completed an initial questionnaire that included demographic information and self-ratings of current physical and mental health status. Hand-held digital audio-recorders were used to record all communication between patients and their prescribing provider. The PI or RA was present to record contextual notes, which provide an important complement for recordings in this setting (e.g. in cases of technological failure or interruptions in the recording) [31]. Providers completed a brief post-visit survey immediately following patient discharge, and patients completed a telephone survey designed to assess satisfaction with the ED visit 24 hours following discharge.

### Measurement and analysis

Table 1 presents the demographic variables that were measured. All audio-recordings were transcribed verbatim and de-identified by a trained transcriptionist. Two coders (RW and DZ) reviewed each transcript to determine whether smoking was discussed and to build a corpus of smoking-related discussions. In all but one encounter, providers initiated communication about smoking as part of the history-taking procedure (e.g. “Do you smoke?”). When patients indicated that they did not smoke, we identified two possible responses from providers: 1) no commentary and 2) positive reinforcement. When patients indicated that they did smoke, we identified three possible responses from providers: 1) no commentary, 2) social commentary, and 3) teachable moments (see Table 2). RW and DZ independently assigned each discussion of smoking to one of these five categories. Only one discrepancy emerged, and it was resolved through discussion.

**Table 1:** **Characteristics of back pain patients visiting an academic emergency department (** ***n*** **= 52)**

***n*** **(%)**			
	***Total***	***Smoker*** **(n = 36)**	***Nonsmoker*** **(** ***n*** **= 16** ***)***
*Patient characteristics*			
Age^*^			
<40	25 (48.0)	20 (55.5)	5 (31.2)
≥40	26 (50.0)	15 (41.6)	11 (68.7)
Sex			
Male	27 (51.9)	18 (50.0)	9 (56.3)^**^
Female	25 (48.1)	18 (50.0)	7 (43.8)
Race			
White	32 (61.5)	20 (55.6)	12 (75.0)
Black/African American	18 (34.6)	14 (38.9)	4 (25.0)
Other	2 (3.8)	2 (5.6)	0 (0)
Marital status			
Married/domestic partnership	18 (34.6)	9 (25.0)	9 (56.3)
Not married	34 (65.4)	27 (75.0)	7 (43.8)
Education			
≤11 years	13 (25.0)	11 (30.6)	2 (12.5)
High school/GED	20 (38.5)	16 (44.4)	4 (25.0)
Some college or greater	19 (36.5)	9 (25.0)	10 (62.5)
Employment status			
Employed	22 (42.3)	18 (50.0)	4 (25.0)
Unemployed	30 (57.6)	18 (50.0)	12 (75.0)
Annual income^*^			
<$10,000	21 (41.2)	16 (45.7)	5 (31.3)
$10,000-$29,999	18 (35.3)	14 (40.0)	4 (25.0)
$30,00-$49,999	7 (13.7)	2 (5.7)	5 (31.3)
≥ $50,000	5 (9.8)	3 (8.6)	2 (12.5)
Insurance			
None	30 (57.7)	22 (61.1)	8 (50.0)
Private	8 (15.4)	4 (11.1)	4 (25.0)
Medicaid/medicare/charity care	14 (26.9)	10 (27.8)	4 (25.0)

^*^Values do not add up to total *n* due to missing data.

^**^Percentages may total more than 100% due to rounding.

**Table 2:** **Non-teachable moment responses to providers’ inquiries about smoking**

***Type of response***	***Example***
**Non-smoker:** ***no response***	N: Ok. Are you a smoker?^*^
	P: Am I a smoker? No. I’ve been clean since, August it will be four years.
	N: Ok. So you quit smoking four years ago? Ok any alcohol or drugs?
	P: No drugs.
**Non-smoker:** ***positive reinforcement***	N: Do you smoke?
	P: No.
	N: Ok.
	P: Quit that two years ago and five months and [twenty-six days.
	N: [Good f-
	N: Good for you! That’s great!
**Smoker:** ***no commentary***	D: Do you smoke?
	P: Yes. Half a [pack a day.
	D: [How much?
	D: Half a pack a day. Do you drink alcohol?
	P: Occasionally.
**Smoker:** ***social commentary***	N: Any drugs?
	P: No ma’am.
	N: Any not-
	P: Tobacco. I guess they consider that a drug. […]
	N: You work in tobacco?
	P: Hum? Yeah. [I do that. I do that. And I do landscaping.
	N: [Tobacco? Ok.
	P: I do basically landscaping. But it is tobacco season, so I am out there helping.
	N: Do you get a discount?
	P: Hum? Uh no [it don’t work like that.
	N: [Do you get a discount?
	N: It doesn’t work like that. ((Both laugh))

^*^See Appendix for transcription conventions.

We then focused further attention on coding “teachable moments” into additional sub-categories that were generated inductively. Categories were refined iteratively to accommodate discrepancies. All instances of teachable moments were then coded by RW and DZ using the following categories: 1) positive reinforcement, 2) encouragement, 3) assessing readiness, and 4) motivating reasons, which were subdivided into 5) medical, 6) family, and 7) economic (see Table 3). These categories were not mutually exclusive; a single encounter might incorporate multiple categories of talk at once. MB, RW, and DZ discussed any remaining discrepancies until a consensus was reached.

**Table 3:** **Teachable moments categories**

***Category and definition***	***Example***
**Positive reinforcement** Provider expresses support for patient’s previous or ongoing attempt to quit smoking.	D: Have you thought about quittin it altogether?
	P: Oh yeah, yeah, I plan on stoppin. Cause she used to smoke but she stopped because of him and
	D: That’s great.
	P: I cut, I cut, I cut down a lot. Like I said, [today I didn’t smoke.
	D: [(Well.) Yeah, well. Extra little bit now, you’re practically there.
**Encouragement** Provider encourages patients to quit smoking but does not provide any advice or tailored feedback.	P: There are times, there are times when I don’t smoke. When (*name*) and (*name*) was here I didn’t smoke for five days. ((laughs))
	D: Um-
	P: So it’s one of things-
	D: [Ok well if you can do it for five days
	P: [(I care about)
	P: It’s not-
	D: It’s not good for you. I’m not telling you anything you don’t know.
	P: Well yeah.
	D: If you can stop, I mean you probably should.
**Assessing readiness** Provider inquires about patient’s willingness to make a quit attempt.	D: Ok. Do you smoke at all?
	P: Yes. Don’t plan on quitting […]
	P: I’m drug and alcohol free except for the medications that I am prescribed.
	D: And the cigarettes.
	P: Well they’re not technically a drug.
	D: Nicotine is.
	P: I don’t believe it.
	D: Ok.
	P: That drug I’m not yet to let go of.
	D: Ok. Alright, well I encourage you to consider it when you’re ready. Ok?
	P: Yes sir.
**Motivating reasons: medical** Provider discusses the health consequences of smoking.	N: Um I encourage you to quit because it causes every known cancer out there in addition to bronchitis, pneumonia and lots of stuff.
	P: Yes ma’am.
**Motivating reasons: family** Provider discusses the consequences of smoking for the patient’s family.	N: Ok. Um do you have kids at home?
	P: No I don’t have my kids at home. My kids live with their mother but uh there’s kids there that I look at like my own so.
	N: Ok.
	P: Yeah.
	N: So you don’t need to be exposing them to-
	P: Yeah.
	N: To tobacco and smoke.
	P: Oh when I smoke I go outside the house.
	N: But it’s still on your clothes.
**Motivating reasons: economic** Provider cites the financial burden of smoking to motivate the patient to quit.	D: Good. Do you smoke?
	P: Yeah.
	D: Well that's a big money saver.
	P: I know.

Finally, we performed an independent sample t-test at the 0.05 level to compare average visit length between encounters with smokers that included a teachable moment and encounters with smokers that did not include a teachable moment. Visit lengths were extracted from the audio-recordings and do not include any intervals during which the provider left the room.

## Results

Of the 104 patients approached, 80 (76.9%) agreed to participate in the study. The most common reasons patients cited for declining were being in too much pain and not wanting to be audio-recorded. Six patients dropped out or were excluded before the study procedures could be completed, for a final sample of 74. Of the 40 providers approached, 32 (80%) agreed to participate in the study. Two providers did not have an opportunity to participate because they were not assigned an eligible patient; the final sample of the larger study thus included 30 providers.

This paper focuses on the 52 patient-provider encounters, including 22 distinct providers, during which smoking was discussed. Table 1 shows the patient characteristics. The mean age of patients was 40.4 years (S.D. = 11.8), with a range of 19–68. Roughly 52% of patients were male and 62% were white. A quarter of all patients did not have the equivalent of a high school degree, 58% were unemployed, and 58% had no medical insurance. In general, the demographic characteristics of smokers reflected the characteristics of the overall sample. Providers were mostly white (90%) and female (68%). Eleven of the providers were nurse practitioners, eight were attending physicians, and three were residents. The mean length of the patient-provider encounter was 17.5 minutes (S.D. = 7.1).

**Figure 1:**
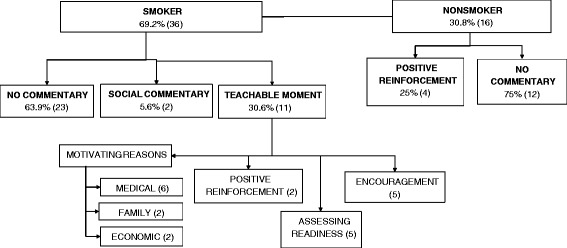
**Patient-provider communication about smoking in the emergency department (** ***n*** **= 52).** Note: teachable moments categories are not mutually exclusive and could be combined within a given patient-provider interaction.

Figure 1 depicts the pathways by which patient-provider communication about smoking was categorized. Roughly two-thirds (*n* = 36) of the patients were smokers. In the majority of cases in which patients identified as smokers (*n =* 23), providers offered no response to this news. For two patients, providers responded with social, non-educational commentary. Providers followed up with a teachable moment in 11 encounters with smokers. The mean visit length for encounters that included a teachable moment was 15.7 minutes (S.D. = 6.6), while the mean visit length for encounters with smokers that did not include a teachable moment was 18.6 minutes (S.D. = 6.8), but this difference was not significant (p = 0.25). Teachable moments ranged from 20 to 143 seconds in duration. An example of a teachable moment involving a male attending physician (D) with 12 years ED experience and a 28-year-old female patient (P) is presented below.

Example 1 Teachable moment

- 1 D: Do you smoke?
- 2 P: Yes.
- 3 D: How much do you smoke?
- 4 P: ‘bout a half a pack to a pack a day.
- 5 D: Do you want to quit?
- 6 P: Yeah.
- 7 D: Ok. How old is your daughter?
- 8 P: Two and a half.
- 9 D: So smoking is not only bad for you.
- 10 P: Yeah.
- 11 D: It’s bad for her.
- 12 D: And if you don’t smoke around her, you’re just 13 bringing it back in on your clothes [and she snuggles 14 up next to you, it’s dangerous to her.
- 15 P: [Yeah.
- 16 P: And she has slight breathing problems too.
- 17 D: Right so if you have asthma, she has asthma and 18 you’re smoking its bad for everyone.
- 19 P: Yeah.
- 20 D: So you really need to do your best to stop.
- 21 D: Have you tried to quit?
- 22 P: Um I have. Just not recently.
- 23 P: Ok. You need to try again. If you can’t do it by 24 yourself, there’s a lot of over the counter stuff. You 25 could try the pill or the gum. You could try the26 patches. If you are not having success doing it on your 27 own, um, go see your doctor at the (*clinic name*) and 28 they can attempt to prescribe you some medication to 29 [help you stop.
- 30 P: [Yeah.
- 31 D: It’s super important both for your health and your 32 daughter’s health that you stop.
- 33 P: Yeah.

Here, the physician employs several strategies to create a teachable moment for smoking cessation counseling. First, he assesses the patient’s readiness by inquiring about whether she wants to quit (line 5; *Assessing Readiness*). Having established that she is motivated to quit, he draws on social information acquired earlier in the visit to deploy a motivational link between the patient’s desire to quit smoking and the health consequences of smoking for the patient’s daughter (lines 7–18; *Family*). On line 12, the physician anticipates a possible defense against the ill effects of secondhand smoke by noting that even if the patient does not smoke around her daughter, there may still be negative repercussions (i.e. third-hand smoke). When the patient provides additional information regarding her daughter’s health risks (line 16), the physician upgrades “slight breathing problems” to “asthma” and emphasizes that smoking has negative medical implications for both the patient and her daughter (line 17-18; *Medical*). Finally, the physician encourages the patient to quit (line 23; *Encouragement*) and mentions concrete resources that may help her in doing so (lines 24–29). The duration of this interaction was one minute.

Example 1. can be contrasted with the two examples below, which we classify as missed opportunities for smoking cessation counseling.

Example 2: Missed opportunity

- 1 D: Do you smoke?
- 2 P: I do. About half a pack a day.
- 3 D: Ok. Do you drink alcohol?
- 4 P: No.
- 5 D: Illegal drugs?
- 6 P: No.
- 7 D: Ok.
- 8 D: I just have to ask these questions.
- 9 P: I know.

In Example 2, an interaction between a male attending physician with 4 years ED experience and a 43-year-old male patient, the patient indicates that he is a smoker but does not use alcohol or illegal drugs. The physician responds with an explanation for this line of questioning (line 8), which suggests that he may feel that these questions are intrusive. The physician does not offer any uptake on the patient’s admission of smoking.

Example 3. Missed opportunity

- 1 D: Are you a smoker?
- 2 P: Yeah.
- 3 D: How much do you smoke?
- 4 P: About a pack a day.
- 5 D: Ok. And how many years have you smoked a pack a 6 day?
- 7 P: Well actually I ain’t started back less than a year. I 8 quit for twenty years.
- 9 D: What. You quit for how many years?
- 10 P: Twenty years.
- 11 D: That’s amazing. And then you just started back?
- 12 P: Yeah.
- 13 F: Nut.
- 14 D: Did you have more stress? Or-
- 15 P: Yeah.
- 16 D: So, so, you’re about a pack a day? Ok. Um do you 17 drink alcohol as well?
- 18 P: Mm hm.

In Example 3, an interaction between a female resident with less than 1 year ED experience and a 44-year-old male patient, the resident expresses amazement that the patient has recently started smoking again after a 20 year hiatus (lines 9-11). This sentiment is echoed by a friend of the patient, who has accompanied him to the ED (line 13). While the resident inquires about the circumstances that led to this change (line 14), she does not capitalize on the opportunity to inquire about the patient’s motivation to quit or to offer resources and/or counseling.

## Discussion

This study characterized patient-provider communication about smoking in the ED within a sample of 52 ED visits for back pain. As in prior research [28], providers in this study typically introduced smoking as a topic of conversation while gathering patients’ medical history. Providers missed opportunities to offer online smoking cessation counseling approximately 70% of the time. In 11 encounters, providers capitalized on the opportunity to offer brief advice about smoking. We identified four primary strategies for creating teachable moments for smoking cessation counseling: positive reinforcement, encouragement, assessing readiness, and offering concrete motivating reasons by linking to patients’ specific medical, family, or economic situations.

Although most research on patient-provider communication about smoking has focused on the primary care setting [7], ED visits represent an important opportunity for patients to receive valuable counseling in health behavior change, and may be particularly beneficial for patients who are uninsured and/or do not have a primary care provider [9,23], which was the case for the majority of the study sample. While counseling in the ED alone is unlikely to be effective as a smoking cessation strategy, the concept of “ED-initiated tobacco control” is promising [9]. The teachable moments documented in this study illustrate what the first step in a multi-stage process might look like. Unfortunately, as in Vokes et al.’s audio-recorded study of patient-provider communication about smoking in the ED, few smoking discussions extended past an initial screening inquiry to offer advice to quit and supportive resources for doing so [27].

In the 11 encounters in our sample that included teachable moments, providers employed multiple strategies, combining general advice with motivation tailored to the patient’s particular circumstances. Contrary to research conducted by Crabtree et al., which found that the length of primary care medical visits is significantly longer when smoking is discussed [46], we did not identify a significant difference between the length of ED encounters that included a teachable moment for smoking cessation counseling and those that did not. Although our small sample size suggests that these results should be interpreted cautiously, it is interesting to note that the encounters that included a teachable moment were actually shorter, by an average of 2.9 minutes. This suggests that smoking cessation counseling need not impose a substantial temporal burden on ED providers. Furthermore, teachable moments for smoking cessation counseling occurred during the history-taking portion of the ED encounter, prior to discussions of treatment. Therefore, it is not simply the case that providers offered smoking cessation counseling if time remained at the end of the visit.

This study has several limitations. First, patients and providers may have changed their communication behavior as a result of being observed. However, the Hawthorne effect was likely minimized because neither patients nor providers were aware that we were examining communication about smoking. Second, our sample size was too small to identify meaningful patterns in the data. Moreover, the study was conducted with back pain patients in a single academic ED. Although back pain is a common, representative ED complaint, further research is necessary to determine whether the presence of teachable moments within the ED encounter is associated with specific patient, provider, or institutional characteristics. Furthermore, we did not examine non-verbal communication and other contextual cues, which may help to account for why some providers did not ask about smoking or pursue smoking cessation counseling. Finally, more work is necessary to determine the specific mechanisms by which teachable moments might facilitate health behavior change [32,38]. Teachable moments may be less successful at facilitating change without the provision of supportive resources and follow-up [47].

Despite these limitations, this study contributes valuable descriptive information about teachable moments and missed opportunities for smoking cessation in the ED. The analysis of transcribed patient-provider communication is an important strength of the study because few studies have examined audio-recordings of live communication about smoking, particularly in the ED. Other studies have used methodological approaches that may overestimate the duration of counseling [46]. The concept of teachable moments offers one possible framework for how a 5A’s approach to smoking cessation counseling might be incorporated into ED encounters in the course of routine care. Therefore, this mixed-methods investigation of naturalistic patient-provider communication makes an important contribution to the smoking cessation literature.

## Conclusions

This study extends prior research on patient-provider communication and teachable moments for health behavior change by applying the concept to the ED encounter. Patient-provider communication in the ED is beset by many challenges, including long wait times, frequent interruptions, time constraints, privacy concerns, and a lack of continuity in care [29-31]. Most providers in this study did not offer smoking cessation counseling when patients indicated that they were smokers. Nevertheless, our findings suggest that teachable moments for smoking cessation counseling may occur with a minimal investment of ED resources.

This study offers support for framing the ED as a viable context for teachable moments for smoking cessation counseling. Asking patients whether they are interested in quitting, providing encouragement and positive reinforcement, and creating motivational links to enhance smoking cessation efforts may be possible without imposing an undue burden on limited provider resources. While some providers possess the skills and resources necessary to incorporate teachable moments into ED encounters, others would likely benefit from further training on effective strategies to promote smoking cessation.

## Appendix. Transcription conventions

N: Nurse practitioner speech

D: Physician speech

P: Patient speech

F: Friend or family member speech

[Denotes overlapping speech

[…] Transcript excerpted

( ) Uncertain content

(*name*) Person or place removed for anonymity

(( )) Nonverbal action

## Abbreviation

ED: Emergency department
